# Impacts of Agricultural Practices on Insecticide Resistance in the Malaria Vector *Anopheles arabiensis* in Khartoum State, Sudan

**DOI:** 10.1371/journal.pone.0080549

**Published:** 2013-11-18

**Authors:** Sara A. Abuelmaali, Arwa H. Elaagip, Mohammed A. Basheer, Ehab A. Frah, Fayez T. A. Ahmed, Hassabelrasoul F. A. Elhaj, Osama M. E. Seidahmed, David Weetman, Muzamil Mahdi Abdel Hamid

**Affiliations:** 1 Department of Medical Entomology, National Public Health Laboratory, Federal Ministry of Health, Khartoum, Sudan; 2 Department of Parasitology and Medical Entomology, Faculty of Medical Laboratory Sciences, University of Khartoum, Khartoum, Sudan; 3 Tropical Medicine Research Institute, National Center for Research, Khartoum, Sudan; 4 Department of Vector Biology, Liverpool School of Tropical Medicine, Liverpool, United Kingdom; 5 Department of Molecular Biology, Institute of Endemic Diseases, University of Khartoum, Khartoum, Sudan; National Institute for Communicable Diseases/NHLS, South Africa

## Abstract

**Background:**

Agricultural pesticides may play a profound role in selection of resistance in field populations of mosquito vectors. The objective of this study is to investigate possible links between agricultural pesticide use and development of resistance to insecticides by the major malaria vector *Anopheles arabiensis* in northern Sudan.

**Methodology/Principal Findings:**

Entomological surveys were conducted during two agricultural seasons in six urban and peri-urban sites in Khartoum state. Agro-sociological data were collected from 240 farmers subjected to semi-structured questionnaires based on knowledge attitude and practice (KAP) surveys. Susceptibility status of *An. arabiensis* (n=6000) was assessed in all sites and during each season using WHO bioassay tests to DDT, deltamethrin, permethrin, Malathion and bendiocarb. KAP analysis revealed that pesticide application was common practice among both urban and peri-urban farmers, with organophosphates and carbamates most commonly used. Selection for resistance is likely to be greater in peri-urban sites where farmers apply pesticide more frequently and are less likely to dispose of surpluses correctly. Though variable among insecticides and seasons, broad-spectrum mortality was slightly, but significantly higher in urban than peri-urban sites and most marked for bendiocarb, to which susceptibility was lowest. *Anopheles arabiensis* from all sites showed evidence of resistance or suspected resistance, especially pyrethroids. However, low-moderate frequencies of the L1014F *kdr* allele in all sites, which was very strongly associated with DDT, permethrin and deltamethrin survivorship (OR=6.14-14.67) suggests that resistance could increase rapidly.

**Conclusions:**

Ubiquitous multiple-resistance coupled with presence of a clear mechanism for DDT and pyrethroids (*kdr* L1014F) in populations of *An. arabiensis* from Khartoum-Sudan suggests careful insecticide management is essential to prolong efficacy. Our findings are consistent with agricultural insecticide use as a source of selection for resistance and argue for coordination between the integrated vector control program and the Ministry of Agriculture to permit successful implementation of rational resistance management strategies.

## Introduction

 Malaria constitutes a major public health problem in Khartoum state, leading to about 300,000 cases and five hundred deaths each year [1,2]. Both urban and peri-urban agricultural areas have been expanded in response to high demand for accommodation and food. However, the relative rate of expansion varies between the state’s districts, due to differences in landscape and the livelihoods of the local community [3]. The WHO recommends large-scale distribution of insecticide-treated nets (ITNs) to control malaria transmission. Recently, concerted international efforts have been devoted to distribute ITNs and also to increase Indoor Residual Spraying (IRS), which has contributed to a major reduction in disease burden in sub-Saharan Africa [4,5]. Likewise ITNs and IRS, are the main vector control strategies for malaria in Sudan [6]. 

In contrast to wider malaria control practice, the main vector control intervention in Khartoum state is larval control using the organophosphate insecticide Temephos, particularly in urban areas [1,2,7,8]. On the other hand environmental management through weekly drying of irrigation canals (intermittent irrigation) is more common in the peri-urban areas of the state [2,3]. In addition space spraying of insecticides and IRS is occasionally used when mosquito densities increase or if a malaria outbreak is suspected. Despite ongoing scale-up in other parts of Sudan coverage with ITNs is still very low in Khartoum [2,3,9].

Resistance of mosquito vectors to insecticide remains one of the major challenges facing malaria control programs [5]. Resistance to pyrethroids and DDT in *An. gambiae* s.s. and *An. arabiensis*, two important African malaria vectors, is particularly widespread [10,11,12]. Pyrethroids and DDT both target a voltage-gated sodium channel, and substitutions of the leucine residue at codon 1014 (L1014F and L1014S) can lead to target site insensitivity known as knock down resistance (kdr). In *An. arabiensis* populations the L1014F allele has been found widely across many eastern and western African countries [6,13,14,15,16].

Owing to pyrethroid and DDT resistance and cross-resistance,, carbamate and organophosphate insecticides, which have a different mode of action, represent important alternatives for IRS.

And are increasingly used in sub-Saharan Africa as a replacement to pyrethroids [17]. Organophosphate resistance has been documented in *An. arabiensis* from Sudan and neighboring Ethiopia [18,19,20], but available studies suggest predominant susceptibility to carbamates [18,20]. Nevertheless, more information on the susceptibility levels of populations of *An. arabiensis* to carbamates and organophosphates is required. In addition the strength of association of *kdr* with DDT and pyrethroid cross-resistance phenotypes requires further exploration[21], because metabolic resistance mechanisms may also be associated with pyrethroids resistance in *An. arabiensis* [22].

Elucidating the susceptibility status of local populations of An. arabiensis to insecticides is therefore essential, particularly in urban settings that may be influenced by small-scale landscape and livelihood variations [3]. The aim of this study was to investigate the effect of habitat type (urban and peri-urban) and agricultural season (summer and winter) on insecticide resistance in An. arabiensis in Khartoum state, Sudan to investigate the hypothesis that agricultural pesticide use will favour resistant phenotypes.

## Materials and Methods

### Ethics statement

Ethical approval for the study was granted by the Ethical Review Committee of the Ministry of Health, Sudan (2010). The objectives and procedures of the study were explained to the local health authorities and to the Khartoum malaria control program. Agreement for the use of data from any farmer recruited to fill the questionnaire was obtained. Field studies did not involve endangered or protected species.

### Study sites

**Figure 1:**
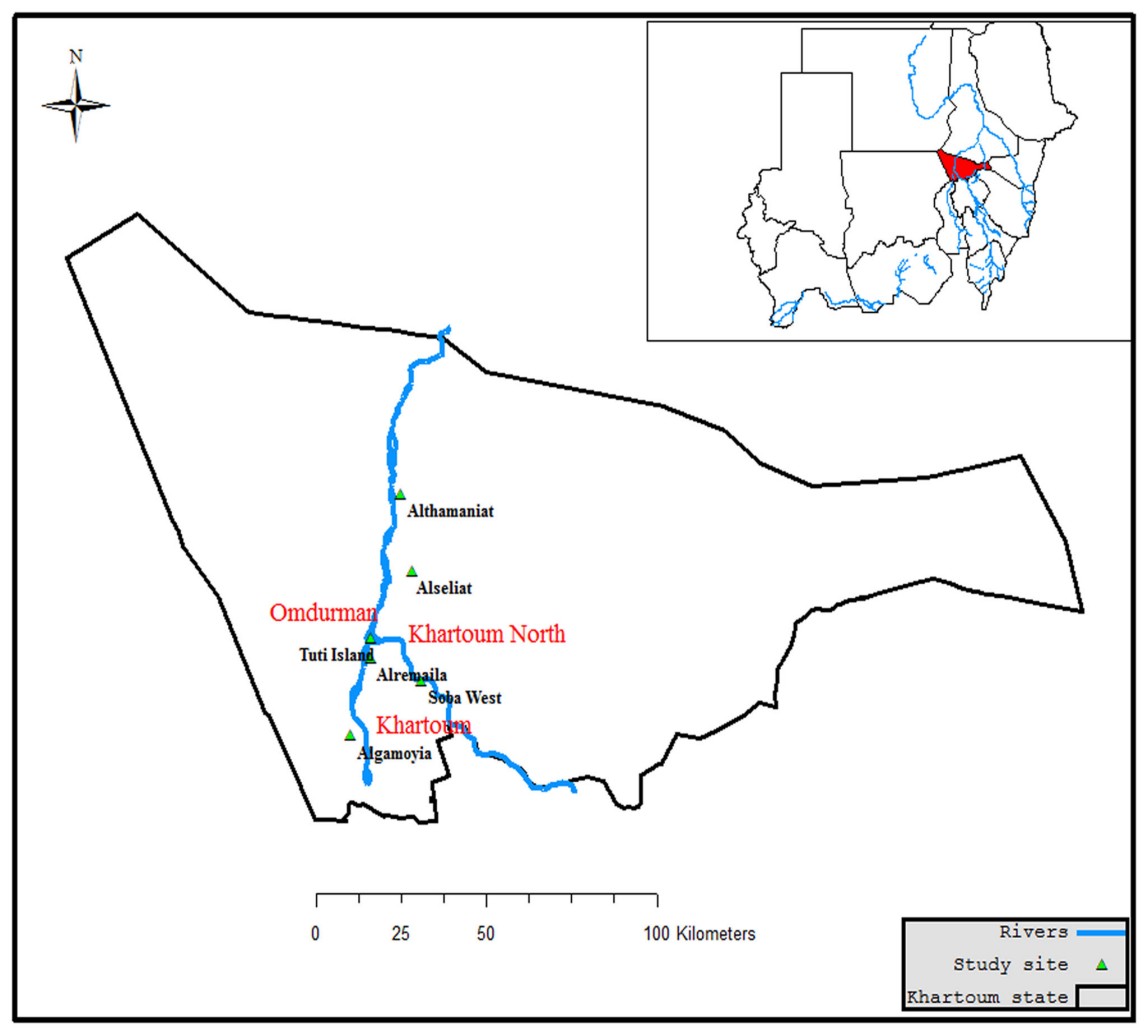
Map showing the study sites in Khartoum state, Sudan including urban sites (Soba West, Alremaila, Tuti island) and peri-urban sites (Althamaniat, Algamoyia, Alseliat).

Khartoum state lies in a poor savannah region, characterized by a short rainy season (July to September), winter season (October to March) and summer season (April to July). The total area of the state is 28000 km^2^; it is divided by the Blue Nile, White Nile and River Nile into three greater administrative areas: Khartoum North, Khartoum and Omdurman. These areas differ in their topography, agriculture and socioeconomic activities. Agricultural activities (mainly along River Nile banks) are the main source of mosquito breeding sites in the River Nile bank areas. Entomological cross-sectional surveys were carried out in Khartoum in six sites categorized as urban and peri-urban where agricultural schemes were found. The urban sites are: Soba West (32°38'14.74"E, 15°31'3.49"N), Alremaila (32°30'16.03"E, 15°34'24.44"N), Tuti island (32°30'20.69"E, 15°37'13.69"N), and the peri-urban: Althamaniat (32°34'52.18"E, 15°57'44.50"N), Algamoyia (32°26'56.48"E, 15°23'14.08"N), Alseliat (32°36'47.22"E, 15°46'41.67"N) (Figure 1). The entomological surveys were conducted during two agricultural seasons: winter (November 2010- March 2011) and summer (April - May 2011). Malaria transmission in the area is unstable and highly seasonal [1]. The primary economic activity of local people in both study sites is farming.

### Mosquito collections and morphological identification

Larvae and pupae of *Anopheles* mosquitoes were collected by dipping from a range of different breeding sites to represent the diversity of the mosquito populations in each study area. Samples were transported to the reference laboratory of mosquito rearing in the Department of Medical Entomology, Federal Ministry of Health, Sudan then reared to adults under standard insectary conditions (25±2°C, 70-80% relative humidity). Adult *Anopheles* mosquitoes were identified to species according to the morphological key [23]. 

### Insecticide susceptibility tests

Susceptibility tests were conducted according to standard WHO bioassay protocol. Insecticide impregnated papers with discriminating concentrations of: DDT (4%), permethrin (0.75%), deltamethrin (0.05%), malathion (5%) and bendiocarb (0.1%) were used. A laboratory susceptible strain of *An. arabiensis* -Dongola colony-was used as a reference colony to test the efficacy of the impregnated papers. For each insecticide five batches of twenty (2-3 day-old sugar-fed) females were exposed to impregnated paper for one hour. Controls included batches of mosquitoes from each site exposed to untreated papers. The number “knocked-down” was recorded at different time intervals (10, 15, 20, 30, 40, 50, and 60 minutes) and after 60 min exposure mosquitoes were transferred into holding tubes and provided with cotton wools soaked with a 10% sucrose solution. Mortalities were recorded 24 hours post-exposure [24]. Dead and surviving mosquitoes from each bioassay were kept separately in eppendorf tubes over silica gel for subsequent molecular analysis.

### Knowledge, Attitude and Practice (KAP) surveys of agricultural pesticide use

To obtain information on the use of pesticides among farmers, KAP surveys were carried out in urban and peri-urban study sites. A sample of 240 farmers was recruited to answer the questionnaires, 40 from each site. The questionnaire was pre-tested in a village not used as a study site. 

### DNA extraction, molecular identification and *kdr* mutations detection

 Sub-samples of dead and live mosquitoes from each insecticide×season×study site combination were selected randomly. Genomic DNA was extracted from single mosquitoes following [25], re-suspended in 200 μl of 1X TE-buffer and stored at -20°C. Molecular identification of *An. arabiensis* was carried out using the PCR diagnostic [26]. A total of 250 female mosquitoes from DDT, permethrin and deltamethrin bioassays were genotyped for *kdr* 1014 alleles using allele-specific PCR assay [27,28]. 

### Statistical analysis

For comparability with other studies, the resistance status of mosquito samples was classified according to the WHO criterion (2013) [24] accordingly, mortality rate ≥98% = susceptible; 90-97% = suspected/potential resistance; and <90% = resistant. Generalized Linear Models (GLM) with a Poisson log linear link function was run in SPSS 20 to test the effect of different factors and their interaction on bioassay mortalities. Chi-square contingency tests were used to test the association of *kdr* with bioassay survivorship for each insecticide, with heterogeneity chi-square tests used to compare association among insecticides. Binomial confidence intervals for bioassays and allele frequencies were computed using Javastat, available at http://statpages.org/confint.html. Moreover, Chi-square test was used to determine the significance of the difference between KAP categorical data (urban and peri-urban sites) using SPSS 20 software. A two sided p value < 0.05 was considered statistically significant.

## Results

### Insecticide susceptibility profiles

**Figure 2:**
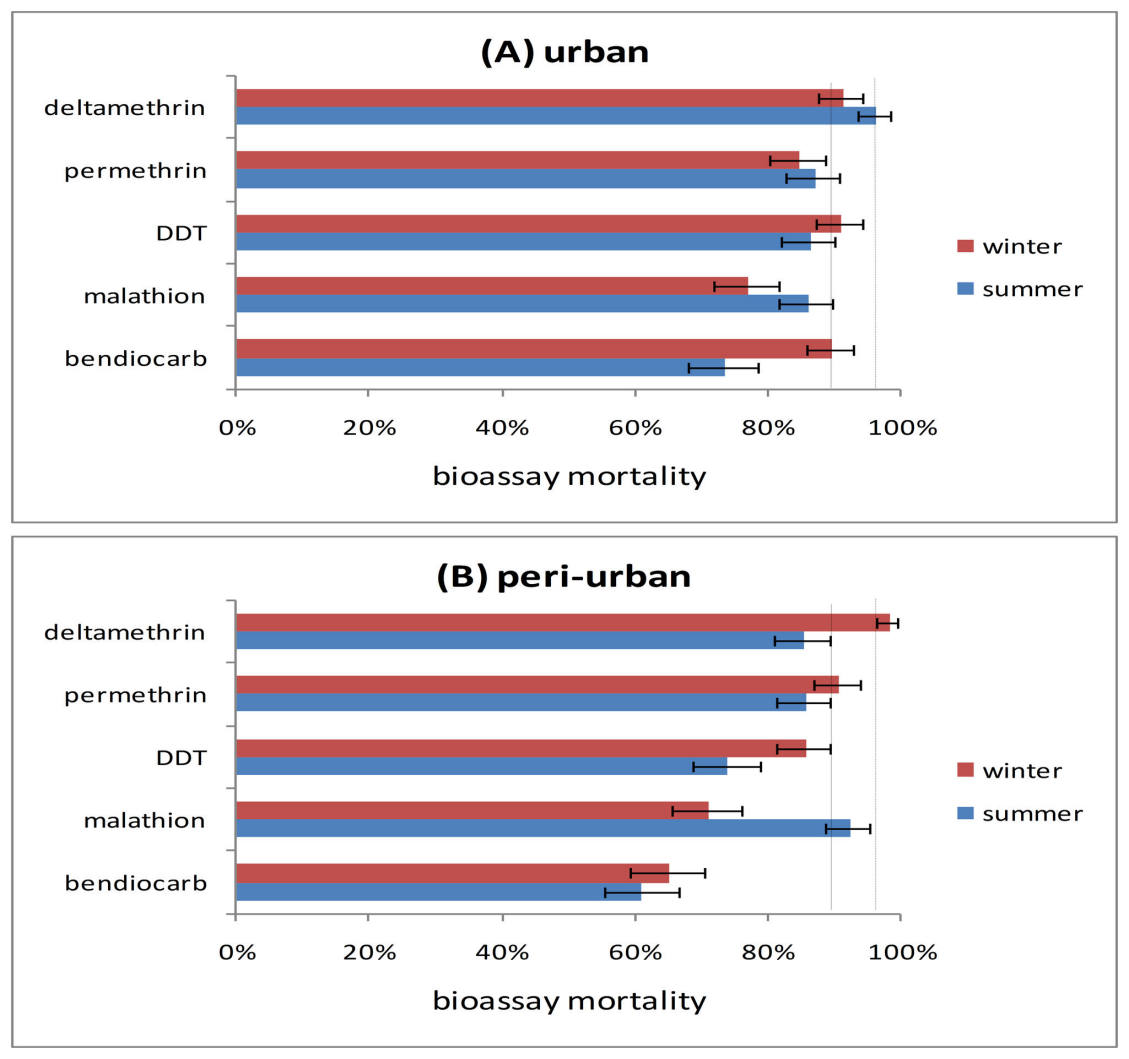
Insecticide resistance bioassay data pooled across sites for mosquitoes collected in each agricultural season in (A) urban and (B) peri-urban sites. Bars show mean mortality with 95% binomial confidence limits. Solid vertical lines show the WHO mortality threshold for definition of resistant mosquitoes; the dashed lines show the threshold for suspected resistance.

A total of 6000 adult mosquitoes reared from larval collection, from both urban and peri-urban sites across two seasons were identified morphologically as *An. gambiae* s.l. A subsample of 100 mosquitoes was subjected to PCR; all proved to be *An. arabiensis*. According to WHO criteria, resistance or potential resistance to all insecticides was detected, with generally highest mortality to deltamethrin and lowest on average to bendiocarb (Figure 2, Table S1, Table S2). GLM analysis indicated that whilst variation among individual sites was not significant, site type was a (marginally) significant factor in determining insecticidal mortality with generally higher mortality in urban than peri-urban sites, but this effect was not consistent across all insecticides (Figure 2, Table 1). In contrast bendiocarb mortality was lower in peri-urban samples with high variability among individual sample sites.

**Table 1:** Generalized linear model testing the effects of insecticide, season, site type (urban or peri-urban) and site (nested within site-type) on bioassay mortality.

**Model terms included**	**χ^2^**	**d.f.**	**P-value**
insecticide	35.5	4	3.7 ×10^-7^
site type	6.5	1	0.011
site(site type)	9.2	4	0.057
insecticide×season	15.7	5	0.008
insecticide × site type	12.1	4	0.016

χ^2^=Chi-square value

d.f=degree of freedom

There was no overall effect of agricultural season on bioassay results with a significant season × insecticide interaction (Table 2) driven by seasonal variation in malathion and bendiocarb though the direction of the seasonal effect differed (Figure 2, Table 3). None of the terms in the GLM were significant for DDT or the two pyrethroids.

**Table 2:** Generalized linear model testing the effects of season, site type (urban or peri-urban) and site (nested within site-type) on bioassay mortality for each insecticide.

**Model term**	**bendiocarb**	**malathion**	**DDT**	**permethrin**	**deltamethrin**
site type	0.0002	NS	NS	NS	NS
Season	0.0149	0.0037	NS	NS	NS
site(site type)	0.0003	NS	NS	NS	NS
season × site type	NS	NS	NS	NS	NS
season × site(site type)	0.0004	NS	NS	NS	NS

NS=not significant

**Table 3:** Summary of KAP agro-sociological data: comparison of studied variables between urban and peri-urban sites.

**Question**	**χ^2^**	**P-value**	**Interpretation**
Educational level	15.78	0.003	Urban more educated than peri-urban
Crop types cultivated	35.67	9 × 10^-8^	Peri-urban greater diversity in crops
Seasonal difference in crop types	3.53	0.31	NS
Use of pesticides	2.30	0.13	NS
Pesticide class(es) used	6.57	0.08	NS
Number of pesticide classes used	1.57	0.46	NS
Source of pesticides used	6.51	0.011	Peri-urban use more from Ministry of Agriculture
Frequency of pesticide application	21.39	9 × 10^-5^	Peri-urban apply more frequently
Pesticide disposal method	8.68	0.003	Correct disposal by urban > peri-urban
Action if pesticides appear to lose efficiency	33.09	1 ×10^-6^	Urban tend to increase application, peri-urban to replace product
Do you think poor use of pesticide causes loss of effectiveness?	3.96	0.14	NS
Perceived seasonality of mosquito density	24.80	0.0001	Urban more seasonal, peri-urban more continuous
Is mosquito protection used?	0	1	NS
Are mosquitoes targeted using agricultural pesticides?	3.63	0.16	NS

NS=not significant

### *Kdr* frequency and resistance-association

Of 289 *An. arabiensis* mosquitoes screened, 250 yielded scorable genotypes for the *kdr* 1014 locus. *Kdr* 1014S was absent from all screened samples but the *kdr* L1014F allele was detected in 124 mosquitoes, almost 90% of which were heterozygotes (Table 4). Owing to relatively low sample sizes, data for seasons were pooled. *Kdr* allele frequencies were very similar in urban (frequency=0.25; 95% confidence intervals 0.2-0.31) and peri-urban sites (frequency=0.30; 95% confidence intervals 0.25-0.36). There was a strong and significant association between possession of the *kdr* allele and bioassay survivorship for DDT, permethrin and deltamethrin in urban and peri-urban sites, with odds ratios between approximately 6 and 15 (Table 4). Whether data were pooled across urban and peri-urban sites to increase power, or treated separately, there was no significant variation in association among the three insecticides. 

**Table 4:** Frequencies of L1014F alleles detected in susceptible and resistant mosquitoes of *An*. *arabiensis* exposed to DDT, permethrin and deltamethrin.

**Site Type**	**Insecticide**	**Phenotype**	**No. per genotype**			**Total**	**Frequency**	**P- value**	**OR**
			**LL**	**LF**	**FF**		**L1014F**		
urban	DDT	Dead	20	6	0	26	0.11	0.0007	7.67
		Alive	0	12	0	12	0.50		
	Permethrin	Dead	24	6	0	30	0.10	2.539 ×10^-7^	12.79
		Alive	0	19	4	23	0.59		
	Delatmethrin	Dead	21	6	0	27	0.11	0.023	8.00
		Alive	0	4	0	4	0.50		
Peri-urban	DDT	Dead	18	11	0	29	0.19	1.605 ×10^-6^	8.83
		Alive	0	15	8	23	0.67		
	Permethrin	Dead	25	5	0	30	0.08	2.192 ×10^-6^	14.67
		Alive	0	12	2	14	0.57		
	Delatmethrin	Dead	18	7	0	25	0.14	0.012	6.14
		Alive	0	7	0	7	0.75		

L=Leucine (wild- type allele); F=Phenylalanine (*kdr* allele); P-value from χ^2^ test of allelic association; OR=odds ratio.

The frequencies were calculated for each insecticide and mosquito status (alive/dead) after exposure.

L1014F represent the *kdr* frequencies

Genotypic odds ratios (ORs) are shown

### Knowledge, Attitude and Practice KAP

**Figure 3:**
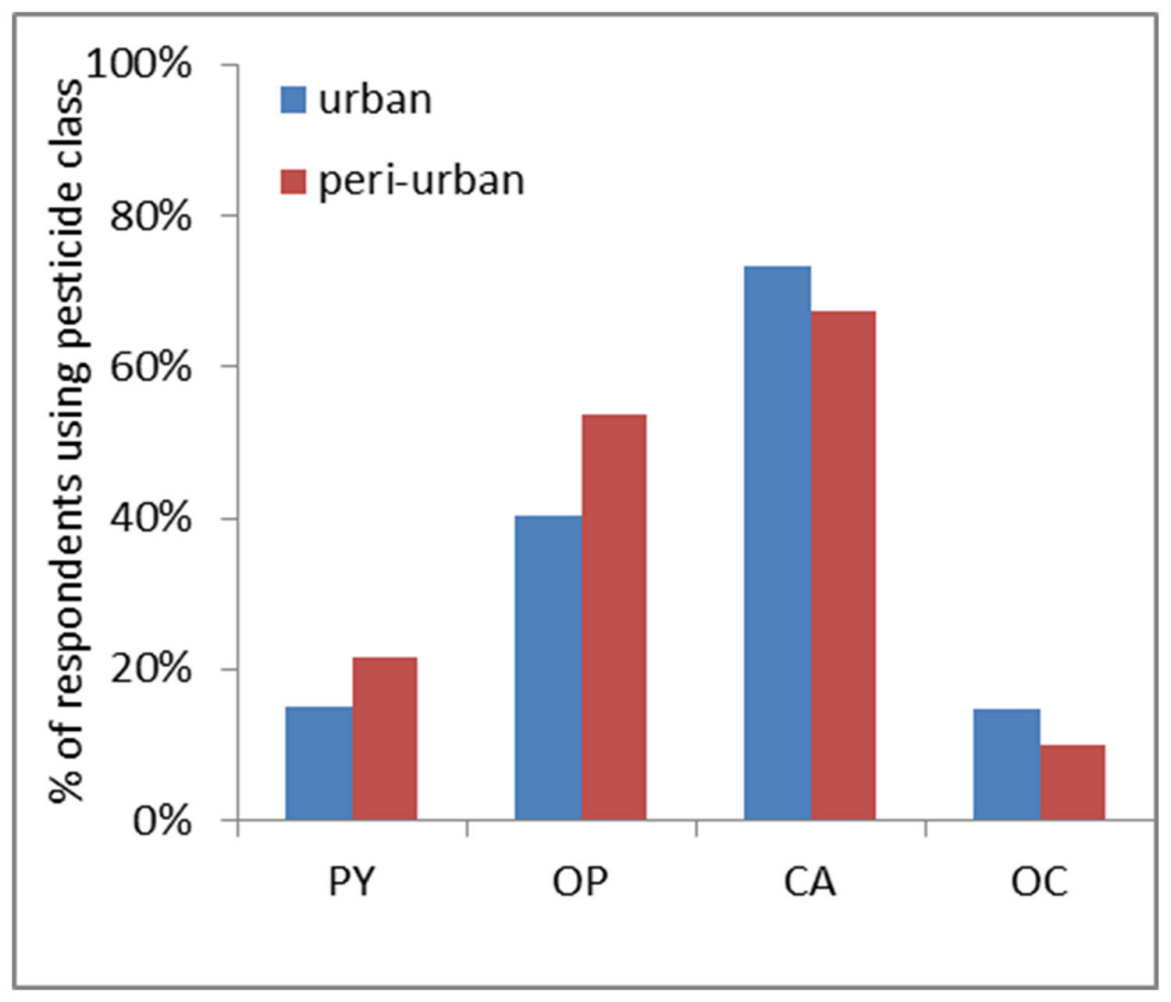
The four pesticides classes used in urban and peri-urban sites during the agro-sociological survey.

The KAP data shows that the most commonly cultivated crops are vegetables in both winter and summer seasons, but that peri-urban farmers grew a greater diversity of other crops, particularly legumes (Table 3). The practice of using pesticides was common among all farmers in different sites, with most sourcing the products from private suppliers, although with a greater tendency of peri-urban farmers to obtain formulations from the Ministry of Agriculture (Table 3). Out of 17 agricultural pesticides used, we focused on the classes used by both farmers and public health authorities. Organophosphates and carbamates were by far the most commonly applied pesticides (Figure 3), although there was no difference between urban and peri-urban farmers in the pesticide classes used or the number applied (Figure 3, Table 3). Peri-urban farmers applied pesticides more frequently and were more likely to replace the product with another class if efficacy was perceived to fall (Table 3). Urban farmers were more likely to dispose of pesticides by an approved method, but there was no significant difference in opinion as to whether poor pesticide application practice impacted efficacy (Table 3).

Farmers most commonly complained of heightened mosquito bites at their homes during the summer season but perceived seasonality of biting was greater in urban homes, with more a continuous mosquito density suggested by peri-urban farmers. The majority of farmers used agricultural products for mosquito control in the home. Breeding sites observed in the urban sites were always road puddles, pools and broken pipelines pools while in peri-urban sites were mainly irrigation canals and hoof prints.

## Discussion

The use of agricultural pesticides may have a profound impact on the development of resistance in the field populations of malaria vectors. This study shows that *An. arabiensis* from urban and peri-urban localities were not fully susceptible to any of the five insecticides tested, though mortality to deltamethrin was generally the highest, and the insecticide for which WHO-defined susceptibility was observed most commonly in individual site ×season combinations.

The ubiquitous detection of suspected resistance to permethrin in all sites is consistent with other studies in Khartoum [3,6] and eastern Africa [20]. However, the prevalence of DDT resistance we detected was higher than detected in previous Sudanese studies [6,29,30]. DDT was banned in Sudan for agricultural use in 1980s, and it is noteworthy that during our KAP agro-sociological surveys we noticed that DDT has been used in agriculture, indicating purchase from illegal markets.

The reduced susceptibility to pyrethroids is of concern for the National Malaria Control Program which depends on efficacious knockdown of mosquitoes by pyrethroids (i.e. ITNs). 

The *kdr* mutation, L1014F, was detected in all urban and peri-urban sites across both summer and winter season. *Kdr* was strongly associated with resistance to pyrethroids and DDT (Table 3) suggesting *kdr* is a primary candidate as a main mechanism underpinning resistance, although metabolic resistance mechanisms may also be involved in pyrethroid resistance in *An. arabiensis* [22] . This finding is in concordance with [31] and [32]. In contrast, previous studies have shown either weak [6] or total absent [29,33] of a significant association between *kdr* mutation and survivorship phenotype indicating that the impact of *kdr* may be very heterogeneous in *An arabiensis*. Of the 17 registered agricultural pesticides applied we focused on those used in agriculture as well as in public health. Organophosphates and carbamates were by far the most commonly used pesticides in agriculture and bendiocarb, the insecticide to which highest levels of resistance were found, particularly in peri-urban sites. Use of the organophosphate malathion by the Khartoum State Malaria Control Program (KSMCP) stopped seven years ago because of development of resistance by mosquitoes vectors (KSMCP, personal communication). Our study confirmed malathion resistance in all urban and peri-urban sites, these results were consistent with other studies in Khartoum [3] . Currently carbamates (e.g. bendiocarb) are not used in public health in Khartoum due to the fact that it has been put as an alternative insecticide for IRS [34]. Furthermore, some studies has raised up the issues of their toxicity and short residual effect if used in impregnation of bed nets [34,35,36]. In our study, it is plausible that the relatively high resistance among the *An. arabiensis* populations in the six sites, particularly the peri-urban sites (Figure 2), may result from accumulation of carbamates from the heavy agricultural use indicated by our KAP survey. Although there was no significant difference in the type of pesticides used between urban and peri-urban sites, farmers in the latter apply pesticides more frequently. Previous studies in Sudan have detected complete susceptibility of *An. arabiensis* populations to bendiocarb from Sennar and Gezira [18,33]. Bendiocarb is not currently used by NMCP but possible selection for resistance by intensive agricultural use is a concern for future public health plans. Further work is required to establish the mechanistic basis of carbamate resistance in *An. arabiensis* and its real magnitude on a large scale basis. 

In conclusion the present study showed at least some evidence for resistance to five insecticides recommended by WHO for malaria control. Pyrethroid resistance appears to be increasing in Khartoum, which was previously an area of relatively high susceptibility in Sudan [18], perhaps due to an increase in the strongly resistance associated *kdr* L1014F allele. With scaling-up of ITNs deployment likely, this is a real concern for malaria control programs in the area and continued monitoring of insecticide resistance is warranted. Although establishing a definitive link between agricultural pesticide application and mosquito resistance is difficult, there is much correlational evidence suggesting that such a link frequently exists [37,38,39,40,41]. Our data also suggest that agricultural pesticide may be causing or enhancing the development of insecticide resistance. We suggest that these results should be considered when devising rational management strategies for integrated insecticide-based control programs that consider both vectors and crop pests. 

## Supporting Information

Table S1 **Mortality rates of *An*.** ***arabiensis* bioassyed to DDT 4%, Permethrin (0.75%), deltamethrin 0.05%, malathion 5 % and Bendiocarb (0.1%) in urban areas in Khartoum, Sudan**.
Show the WHO bioassay test results For mortality rate, percentage, and the resistance status after 24 hours exposure to the five insecticides DDT 4%, Permethrin (0.75%), deltamethrin 0.05%, malathion 5 % and Bendiocarb (0.1%) during winter and summer seasons in urban sites of Khartoum.
* CI=confidence interval, Mortality%: mortality rate 24hours after exposure to each insecticide. ǂR (Resistant), PR (Potential Resistant) and S (Susceptible).
¶Average of five replicates each consists of 20 female mosquitoes.
Number of tested mosquitoes per insecticide per site per season =100.
(DOC)

Table S2 **Mortality rates of *An*.** ***arabiensis* bioassyed to DDT 4%, Permethrin (0.75%), deltamethrin 0.05%, Malathion (5%) and Bendiocarb (0.1%) in peri-urban areas in Khartoum, Northern Sudan**.
Show the WHO bioassay test results For mortality rate, percentage, and the resistance status after 24 hours exposure to the five insecticides DDT 4%, Permethrin (0.75%), deltamethrin 0.05%, malathion 5 % and Bendiocarb (0.1%) during winter and summer seasons in peri-urban sites of Khartoum.
* CI=confidence interval, Mortality%: mortality rate 24hours after exposure to each insecticide. ǂR (Resistant), PR (Potential Resistant) and S (Susceptible).
¶Average of five replicates each consists of 20 female mosquitoes.
Number of tested mosquitoes per insecticide per site per season =100.
(DOC)
