# Correction: Impacts of Agricultural Practices on Insecticide Resistance in the Malaria Vector *Anopheles arabiensis* in Khartoum State, Sudan

**DOI:** 10.1371/annotation/d40e811c-993d-40ec-8294-420402282448

**Published:** 2013-12-10

**Authors:** Sara A. Abuelmaali, Arwa H. Elaagip, Mohammed A. Basheer, Ehab A. Frah, Fayez T. A. Ahmed, Hasabelrasoul F. A. Elhaj, Osama M. E. Seidahmed, David Weetman, Muzamil Mahdi Abdel Hamid

The name of the sixth author was spelled incorrectly. The correct name is: Hasabelrasol F.A. Elhaj. 

