# Supplementary material for: Impacts of Agricultural Practices on Insecticide Resistance in the Malaria Vector Anopheles arabiensis in Khartoum State, Sudan
Source: PLoS One. 2013 Nov 18;8(11):e80549. doi: 10.1371/journal.pone.0080549 (PMC3832379; doi:10.1371/journal.pone.0080549)
Supplement: Table S1 — Mortality rates of An. arabiensis bioassyed to DDT 4%, Permethrin (0.75%), deltamethrin 0.05%, malathion 5 % and Bendiocarb (0.1%) in urban areas in Khartoum, Sudan. Show the WHO bioassay test results For mortality rate, percentage, and the resistance status after 24 hours exposure to the five insecticides DDT 4%, Permethrin (0.75%), deltamethrin 0.05%, malathion 5 % and Bendiocarb (0.1%) during winter and summer seasons in urban sites of Khartoum. * CI=confidence interval, Mortality%: mortality rate 24hours after exposure to each insecticide. ǂR (Resistant), PR (Potential Resistant) and S (Susceptible). ¶Average of five replicates each consists of 20 female mosquitoes. Number of tested mosquitoes per insecticide per site per season =100. (DOC) [file pone.0080549.s001.doc]

**Table S1: Mortality rates of *An. arabiensis* bioassyed to DDT 4%, Permethrin (0.75%), deltamethrin 0.05%, malathion 5 % and Bendiocarb (0.1%) in urban areas in Khartoum, Sudan**

| **Season** | **Study area** | **Insecticide** | **Morality** | | **Resistance statusǂ** |
| --- | --- | --- | --- | --- | --- |
|  |  |  | **(%)** | **Average (95%CI)**¶ |  |
| Winter | Soba West | DDT (4%) | 97 | 19.4 (18.72-20.08) | PR |
|  |  | Permethrin (0.75%) | 97 | 19.4 (18.72-20.08) | PR |
|  |  | Deltamethrin (0.05%) | 98 | 19.6 (18.92-20.28) | S |
|  |  | Malathion (5%) | 77 | | 15.4 (14.72-6.08) | | --- | | R |
|  |  | Bendiocarb (0.1%) | 99 | 19.8 (19.25-20.36) | S |
|  | Alremaila | DDT (4%) | 74 | 16.8 (15.76-17.84) | R |
|  |  | Permethrin (0.75%) | 88 | 14.8 (11.97-17.63) | R |
|  |  | Deltamethrin (0.05%) | 97 | 19.4 (18.29-20.51) | PR |
|  |  | Malathion (5%) | 66 | 15.2 (14.16-16.24) | R |
|  |  | Bendiocarb (0.1%) | 91 | 18.2 (16.58-19.82) | PR |
|  | Tuti Island | DDT (4%) | 92 | 18.4 (17.72-19.08) | PR |
|  |  | Permethrin (0.75%) | 83 | 16.6 (14.72-18.48) | R |
|  |  | Deltamethrin (0.05%) | 79 | 15.8 (13.76-17.84) | R |
|  |  | Malathion (5%) | 78 | 15.6 (13.18-18.02) | R |
|  |  | Bendiocarb (0.1%) | 79 | 15.8 (12.35-19.25) | R |
| Summer | Soba West | DDT (4%) | 87 | 17.4 (12.16-22.64) | R |
|  |  | Permethrin (0.75%) | 88 | 17.6 (14.48-20.72) | R |
|  |  | Deltamethrin (0.05%) | 95 | 19.0 (16.85-21.15) | PR |
|  |  | Malathion (5%) | 96 | 19.2 (17.58-20.82) | PR |
|  |  | Bendiocarb (0.1%) | 45 | 9.0 (9.0-9.0) | R |
|  | Alremaila | DDT (4%) | 85 | 17.0 (17.0-17.0) | R |
|  |  | Permethrin (0.75%) | 93 | 18.6 (17.18-20.02) | PR |
|  |  | Deltamethrin (0.05%) | 98 | 19.6 (18.92-20.28) | S |
|  |  | Malathion (5%) | 75 | 15.0 (13.76-16.24) | R |
|  |  | Bendiocarb (0.1%) | 89 | 17.8 (17.25-18.36) | R |
|  | Tuti Island | DDT (4%) | 87 | 17.4 (15.52-19.28) | R |
|  |  | Permethrin (0.75%) | 80 | 16.0 (14.48-17.52) | R |
|  |  | Deltamethrin (0.05%) | 96 | 19.2 (18.16-20.24) | PR |
|  |  | Malathion (5%) | 87 | 17.4 (15.98-18.82) | R |
|  |  | Bendiocarb (0.1%) | 86 | 17.2 (16.16-18.24) | R |

* CI=confidence interval, Mortality%: mortality rate 24hours after exposure to each insecticide. **ǂ**R (Resistant), PR (Potential Resistant) and S (Susceptible)

¶Average of five replicates each consists of 20 female mosquitoes

Number of tested mosquitoes per insecticide per site per season =100
