# Supplementary material for: Impacts of Agricultural Practices on Insecticide Resistance in the Malaria Vector Anopheles arabiensis in Khartoum State, Sudan
Source: PLoS One. 2013 Nov 18;8(11):e80549. doi: 10.1371/journal.pone.0080549 (PMC3832379; doi:10.1371/journal.pone.0080549)
Supplement: Table S2 — Mortality rates of An. arabiensis bioassyed to DDT 4%, Permethrin (0.75%), deltamethrin 0.05%, Malathion (5%) and Bendiocarb (0.1%) in peri-urban areas in Khartoum, Northern Sudan. Show the WHO bioassay test results For mortality rate, percentage, and the resistance status after 24 hours exposure to the five insecticides DDT 4%, Permethrin (0.75%), deltamethrin 0.05%, malathion 5 % and Bendiocarb (0.1%) during winter and summer seasons in peri-urban sites of Khartoum. * CI=confidence interval, Mortality%: mortality rate 24hours after exposure to each insecticide. ǂR (Resistant), PR (Potential Resistant) and S (Susceptible). ¶Average of five replicates each consists of 20 female mosquitoes. Number of tested mosquitoes per insecticide per site per season =100. (DOC) [file pone.0080549.s002.doc]

**Table S2: Mortality rates of *An. arabiensis* bioassyed to DDT 4%, Permethrin (0.75%), Deltamethrin 0.05%, Malathion (5%) and Bendiocarb (0.1%) in peri-urban areas in Khartoum, Northern Sudan**

| **Season** | **Study area** | **Insecticide** | **Morality** | | **Resistance statusǂ** |
| --- | --- | --- | --- | --- | --- |
|  |  |  | **(%)** | **Average (95%CI)**¶ |  |
| Winter | Soba West | DDT (4%) | 95 | 19 (18.12- 19.88) | PR |
|  |  | Permethrin (0.75%) | 91 | 18.2 (16.36 – 20.04) | PR |
|  |  | Deltamethrin (0.05%) | 98 | 19.6 (18.92 – 20.28) | S |
|  |  | Malathion (5%) | 80 | 15.2 (13.16-17.24) | R |
|  |  | Bendiocarb (0.1%) | 77 | 15.4(10.17-20.63) | R |
|  | Alremaila | DDT (4%) | 79 | 15.8 (14.18 - 17.42) | R |
|  |  | Permethrin (0.75%) | 93 | 18.6 (17.49 -19.71) | PR |
|  |  | Deltamethrin (0.05%) | 99 | 19.8 (19.25 - 20.36) | S |
|  |  | Malathion (5%) | 68 | 13.6 (9.62 – 17.58) | R |
|  |  | Bendiocarb (0.1%) | 65 | 13 (11.48 – 14.52) | R |
|  | Tuti Island | DDT (4%) | 83 | 16.6 (14.93 – 18.27) | R |
|  |  | Permethrin (0.75%) | 88 | 17.6 (16.92- 18.28) | R |
|  |  | Deltamethrin (0.05%) | 98 | 19.6 (18.49-20.71) | S |
|  |  | Malathion (5%) | 69 | 13.8 (12.18- 15.42) | R |
|  |  | Bendiocarb (0.1%) | 53 | 10.6 (4.18- 17.02) | R |
| Summer | Soba West | DDT (4%) | 75 | 15 (8.09-21.91) | R |
|  |  | Permethrin (0.75%) | 92 | 18.4 (16.14-20.66) | PR |
|  |  | Deltamethrin (0.05%) | 100 | 20 (20-20) | S |
|  |  | Malathion (5%) | 94 | 18.8 (17.76-19.8) | PR |
|  |  | Bendiocarb (0.1%) | 61 | 12.2 (9.37-15.03) | R |
|  | Alremaila | DDT (4%) | 68 | 13.6 (6.02-21.18) | R |
|  |  | Permethrin (0.75%) | 85 | 17 (14.68-19.32) | R |
|  |  | Deltamethrin (0.05%) | 88 | 17.6 (16.49-18.71) | R |
|  |  | Malathion (5%) | 95 | 19 (18.12-19-88) | PR |
|  |  | Bendiocarb (0.1%) | 84 | 16.8 (15.76-17.84) | R |
|  | Tuti Island | DDT (4%) | 79 | 15.8 (10.58-21.02) | R |
|  |  | Permethrin (0.75%) | 80 | 16 (14.76-17.24) | R |
|  |  | Deltamethrin (0.05%) | 68 | 13.6 (11.72-15.48) | R |
|  |  | Malathion (5%) | 88 | 17.6 (15.72-19.48) | R |
|  |  | Bendiocarb (0.1%) | 38 | 7.6 (4.61-10.59) | R |

* CI=confidence interval, Mortality%: mortality rate 24hours after exposure to each insecticide. **ǂ**R (Resistant), PR (Potential Resistant) and S (Susceptible)

¶Average of five replicates each consists of 20 female mosquitoes

Number of tested mosquitoes per insecticide per site per season =100
